# Distinct roles for the thioredoxin and glutathione antioxidant systems in Nrf2-Mediated lung tumor initiation and progression

**DOI:** 10.1016/j.redox.2025.103653

**Published:** 2025-04-30

**Authors:** Amanda M. Sherwood, Basma A. Yasseen, Janine M. DeBlasi, Samantha Caldwell, Gina M. DeNicola

**Affiliations:** a Department of Metabolism and Physiology, Moffitt Cancer Center, Tampa, FL, USA; b Cancer Biology PhD Program, University of South Florida, Tampa, FL, USA

**Keywords:** Nuclear factor erythroid 2-related factor 2 (**NRF2**), Kelch-like ECH-Associated protein 1 (**KEAP1**), Glutathione (**GSH**), Glutathione reductase (**GSR**), Thioredoxin (**TXN**), Thioredoxin reductase (**TXNRD1**), Non-small cell lung cancer (**NSCLC**), Reactive oxygen species (**ROS**)

## Abstract

Redox regulators are emerging as critical mediators of lung tumorigenesis. NRF2 and its negative regulator KEAP1 are commonly mutated in human lung cancers, leading to NRF2 accumulation and constitutive expression of NRF2 target genes, many of which are at the interface between antioxidant function and anabolic processes that support cellular proliferation. Nrf2 activation promotes lung tumor initiation and early progression in murine models of lung cancer, but which Nrf2 targets mediate these phenotypes is unknown. Nrf2 regulates two parallel antioxidant systems mediated by thioredoxin reductase 1 (TXNRD1) and glutathione reductase (GSR), which promote the reduction of protein antioxidant thioredoxin (TXN) and tripeptide antioxidant glutathione (GSH), respectively. We deleted TXNRD1 and GSR alone, or in combination, in lung tumors harboring mutations in Kras^G12D^ and Nrf2^D29H^. We found that tumor initiation was promoted by expression of GSR, but not TXNRD1, regardless of Nrf2 status. In contrast, Nrf2^D29H^ tumors, but not Nrf2^WT^, were dependent on TXNRD1 for tumor progression, while GSR was dispensable. Simultaneous deletion of GSR and TXNRD1 reduced initiation and progression independent of Nrf2 status, but surprisingly did not completely abrogate tumor formation. Thus, the thioredoxin and glutathione antioxidant systems play unique roles in tumor initiation and progression.

## 1 Introduction

The transcription factor NRF2 (nuclear factor-erythroid 2 p45-related factor 2) is a stress-responsive transcription factor that regulates the cellular antioxidant response [1,2] and other facets of cellular metabolism [3]. KEAP1 (Kelch-like ECH-associated protein 1) is a negative regulator of NRF2 and, under non-stressed conditions, facilitates the ubiquitin-mediated degradation of NRF2 [4]. Reactive oxygen species and other electrophiles modify reactive cysteine residues on KEAP1, resulting in impaired NRF2 degradation and translocation of NRF2 into the nucleus [5,6], thereby promoting the expression of cytoprotective genes [2,[7], [8], [9], [10], [11], [12], [13], [14]].

The two main antioxidant systems regulated by NRF2 are the glutathione (GSH) [2,7,[15], [16], [17], [18], [19]] and the thioredoxin (TXN) systems [7,[19], [20], [21]]. The GSH system consists of the tripeptide antioxidant glutathione, which is used by glutathione peroxidases (GPXs) [22,23] in the defense against lipid peroxides [22,24,25]. The pool of GSH is maintained by both de novo synthesis and by glutathione reductase (GSR) [16], which regenerates GSH from its oxidized form GSSG. The TXN system is comprised of peroxiredoxins (PRXs) [26], which are the main defense mechanism against hydrogen peroxides [27,28]. Oxidized PRXs are reduced by the protein antioxidant thioredoxins (TXNs) [26,29], which are subsequently reduced by thioredoxin reductases (TXNRDs) [9,28,30]. Both systems overlap in function and targets. GSR and TXNRD1 reduce disulfide bonds and convert cystine (Cys_2_) to cysteine (Cys) [[31], [32], [33], [34]]. The TXN system can also compensate for GSH deficiency, suggesting semi-redundant roles for these systems [32]. Despite their overlap, these redox systems have distinct ROS-scavenging mechanisms [[35], [36], [37]] and differentially regulate cell signaling [[37], [38], [39], [40]].

Genetically engineered mouse models have revealed the complex role of NRF2 in tumor initiation and progression. In some contexts, NRF2 activation can prevent tumor formation [[41], [42], [43]] or have no impact on tumor formation but provide an advantage in response to platinum therapy [44] or protect against EGFR inhibition [45]. In contrast, other studies have described an oncogenic role for Nrf2 in Kras^G12D^-mediated lung tumor initiation and progression [14,41,[46], [47], [48], [49], [50], [51], [52], [53]]. We generated a conditional allele of Nrf2^D29H^ [41], a DLG motif mutant that cannot be properly ubiquitinated and degraded by the KEAP1/CUL3 complex. In combination with the Kras^G12D/+^ model of early lung adenocarcinoma [54], we reported that Nrf2^D29H^ promoted both tumor initiation and early progression to the adenoma stage [41]. However, the roles of the Nrf2-regulated antioxidant systems in mediating these phenotypes were unknown. We focused on TXNRD1 and GSR, the key mediators of the thioredoxin- and glutathione-dependent systems, and deleted these enzymes alone or in combination to study their roles in tumor initiation and early progression.

## 2 Results

### 2.1 Nrf2 promotes the expression of GSR and TXNRD1 in murine lung tumors

**Fig. 1:**
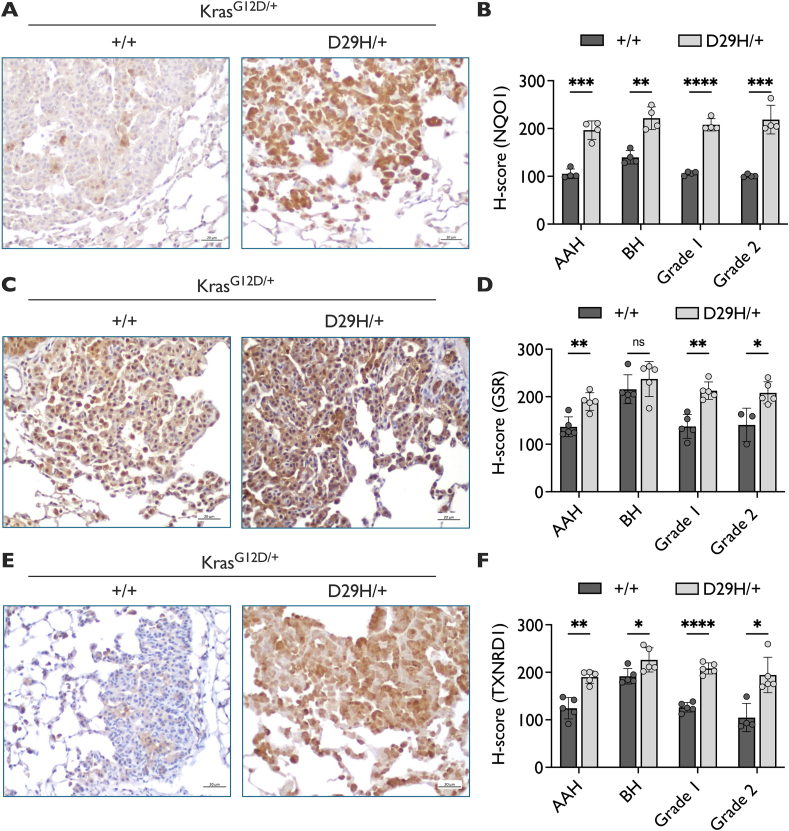
**NQO1, GSR and TXNRD1 expression increase upon Nrf2 activation.** Representative IHC staining for (**A**) NQO1, (**C**) GSR and (**E**) TXNRD1 in Kras^G12D/+^ and Kras^G12D/+;^ Nrf2^D29H/+^ tumors. Scale bars, 20 μm. Graphs depicting the H-score per grade in Nrf2^+/+^ and Nrf2^D29H/+^ tumors for (**B**) NQO1 (**D**) GSR and (**F**) TXNRD1. Nrf2^+/+^ (*n* = 4); Nrf2^D29H/+^ (*n* = 4) in (B); Nrf2^+/+^ (*n* = 5, except where sufficient tumors were available for grade 2 analysis); Nrf2^D29H/+^ (*n* = 5) in (D) and (F). Since grade 3 tumors are rare in the Kras^G12D/+^ model, the H-score analysis per grade was only conducted up to grade 2 tumors. ∗p < 0.05, ∗∗p < 0.01, ∗∗∗p < 0.001, ∗∗∗∗p < 0.0001, ns: non-significant (unpaired *t*-test with Holm–Sidak multiple comparisons test). AAH, alveolar adenomatous hyperplasia; BH, bronchiolar hyperplasia.

We first determined whether Nrf2 activation increased the protein expression of GSR and TXNRD1 in lung tumors by performing immunohistochemical (IHC) staining for the Nrf2 targets NQO1, GSR and TXNRD1 in Kras^G12D^; Nrf2^+/+^ and Kras^G12D^; Nrf2^D29H^ lung tumors. As previously reported, Nrf2^D29H^ increased the expression of NQO1 in tumors (Fig. 1A and B) [41] and also increased the expression of GSR and TXNRD1 (Fig. 1C–F). While bronchiolar hyperplasia had higher expression of these proteins compared to alveolar hyperplasia and grade 1 and grade 2 adenomas, Nrf2^D29H^ promoted their expression across most lesion types and expression remained high across tumor grades (Fig. 1B–D, F). These results indicate that Nrf2 promotes GSR and TXNRD1 expression in Kras^G12D/+^ lung tumors *in vivo*, and suggests these proteins may mediate the effects of Nrf2^D29H^ in promoting initiation and progression.

### 2.2 GSR deficiency impairs lung tumor initiation, but not progression, independent of Nrf2 status

**Fig. 2:**
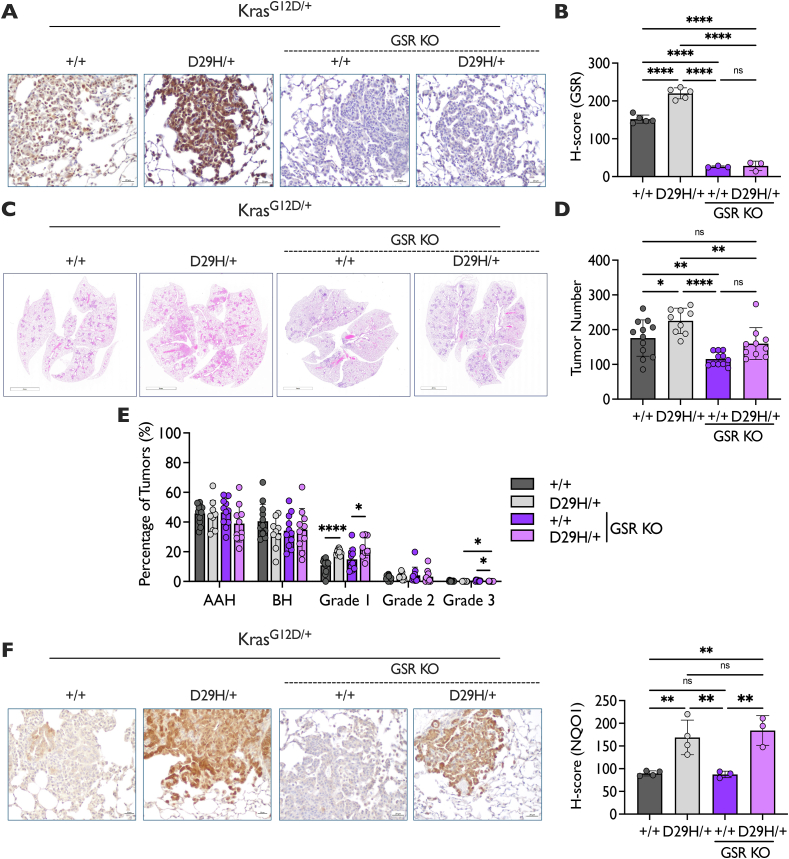
**GSR KO impairs Nrf2^+/+^ and Nrf2^D29H/+^ tumor initiation. (A)** Representative GSR immunohistochemical (IHC) staining of Kras^G12D/+^ and Kras^G12D/+;^ Nrf2^D29H/+^ tumors. Scale bars, 20 μm. **(B)** H-scores for GSR IHC staining. GSR WT (*n* = 5), GSR KO (*n* = 3). ∗∗∗∗p < 0.0001, ns: non-significant (one-way ANOVA with Tukey's multiple comparison's test). **(C)** Representative whole-lung hematoxylin and eosin–stained section. Scale bars, 4000 μm. **(D)** Tumor number per mouse of GSR KO mice (*n* = 11 for Nrf2^+/+^; *n* = 10 for Nrf2^D29H/+^) compared to GSR WT mice (*n* = 12 for Nrf2^+/+^; *n* = 9 for Nrf2^D29H/+^). ∗p < 0.05, ∗∗p < 0.01, ∗∗∗∗p < 0.0001, ns: non-significant (one-way ANOVA with Tukey's multiple comparison's test). **(E)** Distribution of tumor grades across GSR WT (*n* = 12 for Nrf2^+/+^; *n* = 9 for Nrf2^D29H/+^) and GSR KO (*n* = 11 for Nrf2^+/+^; *n* = 10 and Nrf2^D29H/+^) models. ∗p < 0.05, ∗∗∗∗p < 0.0001 (unpaired *t*-test with Holm–Sidak multiple comparisons test). **(F)** Representative IHC staining of NQO1 in Kras^G12D/+^ and Kras^G12D/+;^ Nrf2^D29H/+^ mice. Scale bars, 20 μm. H-scores for NQO1 IHC staining. GSR WT (*n* = 4), GSR KO (*n* = 3). ∗∗p < 0.01, ns: non-significant (one-way ANOVA with Tukey's multiple comparison's test).

Using the Gr1^a1Neu^ allele [55,56] which is a whole-body knockout (KO) allele of GSR, we generated Kras^G12D/+^; Nrf2^+/+^; Gsr^a1Neu/a1Neu^ (GSR KO) and Kras^G12D/+^; Nrf2^D29H/+^; Gsr^a1Neu/a1Neu^ (GSR KO) lung tumors to evaluate the requirement of GSR for tumor initiation and progression. First, we validated the lack of GSR protein by performing immunohistochemical staining for GSR (Fig. 2A and B). Next, we evaluated the influence of GSR on tumor initiation by quantifying tumor number across the genotypes. We found that GSR KO significantly decreased tumor number in both the Kras^G12D/+^; Nrf2^+/+^ and Kras^G12D/+^; Nrf2^D29H/+^ models (Fig. 2C and D). We next examined the impact of GSR on tumor progression by analyzing tumor grade from adenomatous and bronchiolar hyperplasia (AAH and BH, respectively) to tumors from grades 1 (adenoma) to 3 (adenocarcinoma). However, GSR expression did not impact the frequency of the various lung tumor grades, and the increase in grade 1 tumors we previously reported upon Nrf2^D29H/+^ expression [41] was still evident in the absence of GSR (Fig. 2E). We also examined lung tumor burden by grade, which is defined as the percentage of the lung covered by each tumor grade. Interestingly, while GSR loss did not significantly influence grade 1 and 2 tumor burden in the Nrf2^+/+^ model, it did so in the Nrf2^D29H/+^ model (Fig. S1A–B). To test if GSR KO resulted in compensatory induction of Nrf2 and TXNRD1, we performed immunohistochemistry to stain for TXNRD1 and NQO1. However, we found that GSR KO did not alter the expression of these proteins (Fig. 2F, Fig. S2A–B). Overall, these findings indicate that while GSR promotes the formation of lung tumors, GSR is dispensable for the early progression to low-grade tumors, but may contribute to their growth.

### 2.3 TXNRD1 deficiency impairs lung Nrf2^D29H^-dependent tumor progression, but not initiation

**Fig. 3:**
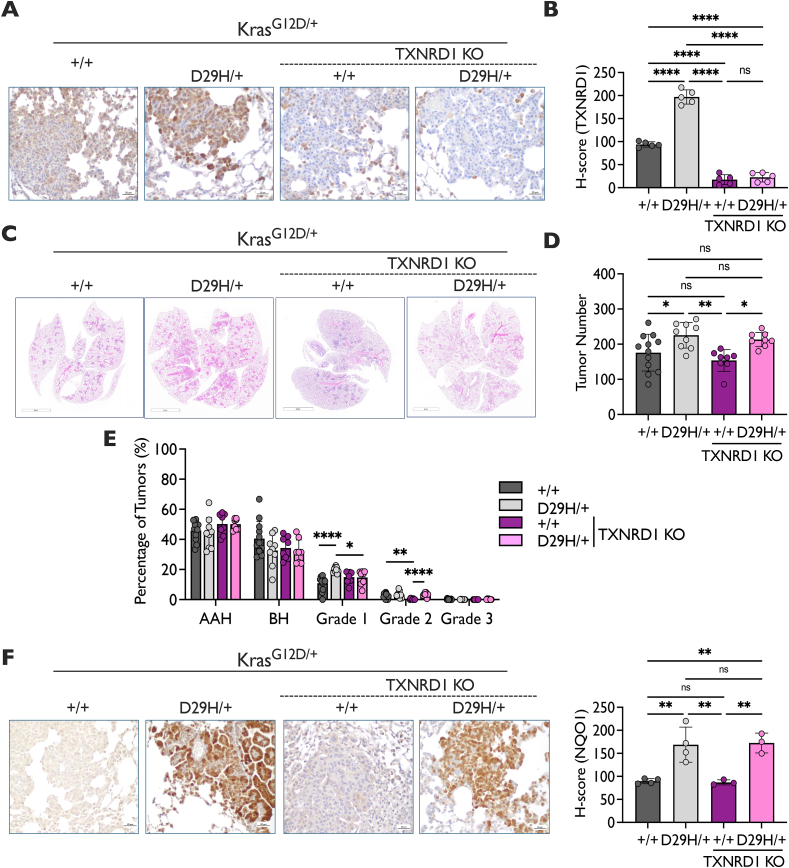
**TXNRD1 KO impairs NRF2^D29H/+^ tumor progression. (A)** Representative TXNRD1 immunohistochemical (IHC) staining of Kras^G12D/+^ and Kras^G12D/+;^ Nrf2^D29H/+^ tumors. Scale bars, 20 μm. **(B)** H-scores for TXNRD1 IHC staining. *n* = 5 mice per genotype. ∗∗∗∗p < 0.0001, ns: non-significant (one-way ANOVA with Tukey's multiple comparison's test). **(C)** Representative whole-lung hematoxylin and eosin–stained section. Scale bars, 4000 μm. **(D)** Tumor number per mouse of TXNRD1 KO mice (*n* = 8 for Nrf2^+/+^; *n* = 8 for Nrf2^D29H/+^) compared to TXNRD1 WT mice (*n* = 12 for Nrf2^+/+^; *n* = 9 for Nrf2^D29H/+^)^.^ N.B. TXNRD1 WT mice are the same as GSR WT mice from Fig. 2D. ∗p < 0.05, ∗∗p < 0.01, ∗∗∗∗p < 0.0001, ns: non-significant (one-way ANOVA with Tukey's multiple comparison's test). (**E**) Distribution of tumor grades across TXNRD1 WT (*n* = 12 for Nrf2^+/+^; *n* = 9 for Nrf2^D29H/+^)^.^ and TXNRD1 KO mice (*n* = 8 for Nrf2^+/+^; *n* = 8 for Nrf2^D29H/+^) models. N.B. TXNRD1 WT mice are the same as GSR WT mice from Fig. 2E. ∗p < 0.05, ∗∗p < 0.01, ∗∗∗∗p < 0.0001 (unpaired *t*-test with Holm–Sidak multiple comparisons test). **(F)** Representative NQO1 IHC staining of Kras^G12D/+^ and Kras^G12D/+;^ Nrf2^D29H/+^ mice. Scale bars, 20 μm. H-scores for NQO1 IHC staining. TXNRD1 WT (*n* = 4), TXNRD1 KO (*n* = 3). ∗∗p < 0.01, ns: non-significant (one-way ANOVA with Tukey's multiple comparison's test).

We next focused on TXNRD1. Using the Txnrd1^flox^ allele [57] to conditionally delete Txnrd1 only in lung cells exposed to cre recombinase, we generated Kras^G12D/+^; Nrf2^+/+^; Txnrd1^Δ/Δ^ (TXNRD1 KO) and Kras^G12D/+^; Nrf2^D29H/+^; Txnrd1^Δ/Δ^ (TXNRD1 KO) lung tumors to evaluate the requirement of TXNRD1 for tumor initiation and progression. We performed immunohistochemical staining of TXNRD1 protein to confirm that TXNRD1 was deleted (Fig. 3A and B). Both TXNRD1 KO models were largely negative for TXNRD1, although there was still some patchy expression largely localized to macrophages and some bronchiolar cells. Unlike what was observed upon GSR loss, we found that deleting TXNRD1 had no impact on tumor number (tumor initiation) in the Kras^G12D/+^, Nrf2^+/+^ or Kras^G12D/+^, Nrf2^D29H/+^ mice (Fig. 3C and D). We then examined the role of TXNRD1 on tumor progression by analyzing tumor grades. TXNRD1 KO abolished the Nrf2^D29H^-mediated increase in grade 1 adenomas (Fig. 3E), but had no effect on Nrf2^+/+^ tumor progression to grade 1 adenomas. Surprisingly, there was a significant increase in the burden of Nrf2^+/+^ alveolar hyperplasia (AAH) and grade 1 tumors upon TXNRD1 KO, which was accompanied by an increase in AAH and grade 1 tumor size (Fig. S1C–D). Because we noticed TXNRD1 KO tumors appeared to be heavily infiltrated by macrophages upon examination of the H&E images, we performed immunohistochemical staining for the macrophage marker F4/80. We found that there was a significant increase in F4/80 staining in TXNRD1 KO tumors compared to all other genotypes, with as much as 40 % of the tumor content being macrophages (Fig. S3D–E). Thus, the increase in burden in Nrf2^+/+^ TXNRD1 KO tumors may be accounted for by the enhanced macrophage infiltration. Like GSR loss, TXNRD1 loss did not affect the expression of the Nrf2 target proteins NQO1 (Fig. 3F) and GSR (Fig. S2C–D), suggesting that inactivating the TXN system does not activate Nrf2 or induce compensatory GSR upregulation. Overall, these findings indicate that although TXNRD1 is dispensable for tumor initiation, TXNRD1 mediates the early tumor progression effects of Nrf2^D29H/+^. Furthermore, the TXN system may play an immunomodulatory role and disruption of this system leads to increased macrophage infiltration.

### 2.4 Simultaneous ablation of GSR and TXNRD1 impairs initiation and progression

**Fig. 4:**
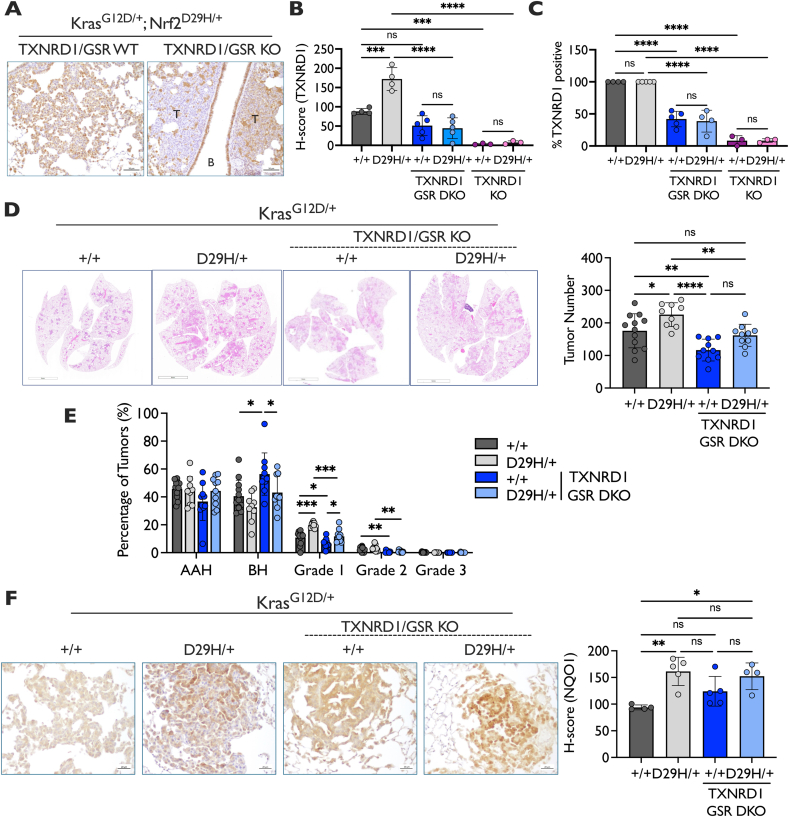
**Dual deletion of GSR and TXNRD1 impairs Nrf2^+/+^ and Nrf2^D29H/+^ tumor formation and progression.** (**A**) Representative TXNRD1 IHC staining of Kras^G12D/+;^ Nrf2^D29H/+^ TXNRD1/GSR WT and DKO tumors depicting positive and negative tumors. Scale bars, 50 μm. B, bronchiole; T, tumor. (**B**) H-score of TXNRD1 expression and (**C**) quantification of percentage (%) of Kras^G12D/+;^ Nrf2^+/+^ and Kras^G12D/+;^ Nrf2^D29H/+^ GSR/TXNRD1 WT (*n* = 4), TXNRD1 KO (*n* = 3) and GSR/TXNRD1 DKO (*n* = 5) tumors per mouse that are TXNRD1 positive. For (C) whole tumors were called as either positive or negative based on TXNRD1 staining. ∗∗∗p < 0.001, ∗∗∗∗p < 0.0001, ns: non-significant (one-way ANOVA with Tukey's multiple comparison's test). (**D**) Representative whole-lung hematoxylin and eosin–stained sections. Scale bars, 4000 μm. Tumor number per mouse of TXNRD1/GSR DKO mice (*n* = 10 for Nrf2^+/+^ and Nrf2^D29H/+^) compared to TXNRD1/GSR WT mice (*n* = 12 for Nrf2^+/+^; *n* = 9 for Nrf2^D29H/+^). N.B. TXNRD1/GSR WT mice are the same as GSR WT mice from Fig. 2D. ∗p < 0.05, ∗∗p < 0.01, ∗∗∗p < 0.001, ns: non-significant (one-way ANOVA with Tukey's multiple comparison's test). (**E**) Distribution of tumor grades across TXNRD1/GSR WT mice (*n* = 12 for Nrf2^+/+^; *n* = 9 for Nrf2^D29H/+^) and TXNRD1/GSR DKO mice (*n* = 10 for Nrf2^+/+^ and Nrf2^D29H/+^) models. N.B. TXNRD1/GSR WT mice are the same as GSR WT mice from Fig. 2E. ∗p < 0.05, ∗∗p < 0.01, ∗∗∗p < 0.001, ∗∗∗∗p < 0.0001 (unpaired *t*-test with Holm–Sidak multiple comparisons test). **(F)** Representative NQO1 immunohistochemical (IHC) staining of Kras^G12D/+^ and Kras^G12D/+;^ Nrf2^D29H/+^ mice. Scale bars, 20 μm. H-scores for NQO1 IHC staining. ∗p < 0.05, ∗∗p < 0.01, ns: non-significant (one-way ANOVA with Tukey's multiple comparison's test). TXNRD1/GSR WT (*n* = 4 for Nrf2+/+ and *n* = 5 for Nrf2^D29H/+^); TXNRD1/GSR KO (*n* = 5 for Nrf2+/+ and *n* = 4 for Nrf2^D29H/+^).

The differential effects on tumor initiation and early progression following GSR and TXNRD1 ablation in Kras^G12D/+^ tumors suggests non-overlapping functions; however, in both models, tumors were still able to form. GSR and TXNRD1 are the only two disulfide reductases in the cytoplasm and concomitant deletion of both would be expected to prevent the reduction of glutathione, protein disulfides, and cystine, and thereby induce lethal oxidative stress. To address the consequence of full disulfide reductase loss in tumors, we generated Kras^G12D/+^; Nrf2^+/+^ and Kras^G12D/+^; Nrf2^D29H/+^ TXNRD1/GSR double KO (DKO) tumors. We performed immunohistochemical staining of TXNRD1 protein to confirm that TXNRD1 was deleted in the DKO model (Fig. 4A–C). Unlike the TXNRD1 KO model, which was largely negative for TXNRD1, the DKO model retained some staining for TXNRD1, although it was less than the TXNRD1 WT model, suggesting some tumors had fully recombined the TXNRD1^flox^ allele (Fig. 4B). We analyzed what fraction of tumors were TXNRD1 positive and found that approximately 40 % of tumors in both the Kras^G12D/+^; Nrf2^+/+^ and Kras^G12D/+^; Nrf2^D29H/+^ models had retained TXNRD1 expression (Fig. 4C). Thus, lung tumors lacking both GSR and TXNRD1 could form, although escape from TXNRD1 recombination was frequent, suggesting loss of both disulfide reductases was disadvantageous.

Like what we observed in the GSR KO model, we found that TXNRD1/GSR DKO significantly reduced tumor number (tumor initiation) in both the Kras^G12D/+^, Nrf2^+/+^ mice and Kras^G12D/+^, Nrf2^D29H/+^ models (Fig. 4D). Interestingly, unlike the TXNRD1 KO model, the TXNRD1/GSR DKO model did not exhibit a significant increase in F4/80 staining, suggesting that GSR expression was required for the alteration in macrophage content upon TXNRD1 loss (Fig. S3E–F). To explore the macrophage phenotype in more detail, we stained for macrophage markers Arg1 and iNOS. However, we only detected positivity for Arg1, suggesting macrophages in these models are immunosuppressive regardless of tumor GSR/TXNRD1 status (Fig. S4).

We next examined the effect of TXNRD1/GSR DKO on tumor progression by analyzing tumor grades. Unlike TXNRD1 KO, which selectively influenced Nrf2^D29H/+^-mediated early progression, TXNRD1/GSR DKO decreased the percentage of grade 1 and grade 2 tumors in both the Nrf2^+/+^ and Nrf2^D29H/+^ models (Fig. 4E). This was accompanied by a decrease in grade 2 tumor size in the Nrf2^+/+^ TXNRD1/GSR DKO mice compared to Nrf2^+/+^ TXNRD1/GSR WT mice (Fig. S1E–F). We performed immunohistochemical staining for the Nrf2 target NQO1 to see if impairing both the GSH and TXN systems resulted in increased Nrf2 activity. Interestingly, there was a modest, although non-significant, increase in NQO1 activation in the Nrf2^+/+^ TXNRD1/GSR DKO tumors (Fig. 4F), suggesting loss of both enzymes may induce mild oxidative stress. Because TXNRD1/GSR DKO livers accumulate protein-glutathione (GSH) adducts, we performed IHC analysis of GSH adducts on TXNRD1/GSR DKO livers compared with our lung models. Interestingly, while accumulation of GSH adducts was detectable in DKO livers, there was no significant difference in their levels across the lung models (Fig. S5A–B). Overall, these data suggest that the GSH and TXN systems have different, although complementary, roles in initiation and progression and that inhibition of both systems impairs tumor formation and progression.

## 3 Discussion

Our finding that the GSH system was more essential for tumor initiation while the TXN system was more essential for tumor progression is consistent with prior work in breast cancer, which found that inhibition of GSH synthesis impaired tumor initiation, but once formed tumors became reliant on the TXN system for growth [58]. However, our study differs from this study in that they inhibited GSH synthesis, whereas we targeted GSH regeneration. Given that our tumors were still capable of GSH synthesis, it is surprising that inhibition of GSH recycling impaired tumor initiation significantly. Because Nrf2^D29H^ still promoted tumor initiation in the absence of GSR, it is likely that this is due to expression of GCLC and GCLM, Nrf2 targets that promote GSH synthesis [17,19]. Moreover, other antioxidant enzymes may compensate for GSR loss. When interpreting results from this study, it is also important to note that GSR activity is also lacking in the tumor microenvironment in addition to the lung tumor cells. Future work targeting GSR KO to only the lung tumor cells, and combining it with inhibition of GSH synthesis will reveal whether inhibition of both GSH synthesis and recycling within the tumor cells abolishes the Nrf2-mediated tumor initiation phenotype.

We found that not only was TXNRD1 essential for Nrf2^D29H^-mediated tumor progression, but TXNRD1 KO tumors also exhibited an increase in macrophage infiltration. One potential explanation for this is ER stress, which induces cytokine production that may promote macrophage recruitment [59]: TXNRD1 is required for protein folding in the ER [60], and ER stress has been linked to glycosylation of cytokines and immune cell recruitment [61]. Secreted TXN also displays cytokine-like properties [62,63], serving as a chemoattractant for various immune cell types [64]. Additional work is needed to understand why macrophage infiltration is increased in TXNRD1 KO tumors, whether these macrophages are protumorigenic or antitumorigenic, and why these macrophages are not present in TXNRD1/GSR DKO tumors.

Tumors lacking both TXNRD1 and GSR could unexpectedly form but it is unclear how this is possible. TXNRD1/GSR null hepatocytes maintain redox homeostasis by shuttling dietary methionine into the transsulfuration pathway to produce Cys for GSH synthesis [65]. However, we previously reported that both healthy mouse lungs and LUAD tumors lack transsulfuration capacity for cysteine synthesis [66] and thus should not be able to sustain their redox state from methionine. Additional research is needed to identify other antioxidant systems or cysteine acquisition mechanisms compensating for the loss of GSR and TXNRD1. Additional work is also needed to understand why, unlike their hepatic counterparts, the TXNRD1/GSR null lung tumor cells do not accumulate protein-glutathione disulfides. It is possible that cells with excessive amounts of these adducts are cleared or rather the tumor cells have a lower basal level of oxidative stress that limits their formation.

Lastly, although we and others have shown that Nrf2 promotes lung tumor initiation and early progression, we previously reported that Nrf2 activation blocks progression to advanced grade adenocarcinomas [41]. Factors such as the timing of NRF2 activation, NRF2 dosage [41], the order of mutations, cell of origin, and NRF2-induced stresses may influence the positive versus negative effects of NRF2. Chronic Nrf2 stabilization has been associated with various metabolic liabilities, including reductive stress [67], glutamate deprivation [14, 68], and CDO1-dependent NADPH deletion [11]. Future work in GEMMs of advanced LUAD progression such as the LSL-Kras^G12D/+^; p53^flox/flox^ model will reveal whether the glutathione and thioredoxin systems play a role in Nrf2-mediated adenocarcinoma suppression. Moreover, they will be critical in clarifying the roles of the GSH and TXN systems in advanced LUAD independently of Nrf2.

## 4 Materials and methods

**Mice**. The housing and breeding of mice in this study adhered to the ethical guidelines and approval of the Institutional Animal Care and Use Committee (protocol #: IS00007922R). Alleles in this study were described previously, including the *LSL-Nfe2l2*^*D29H*^ (MGI: 7327101) [41]; *Txnrd1*^*flox*^ (RRID: IMSR_JAX:028283) [57], *LSL-Kras*^*G12D/+*^ (RRID:IMSR_JAX:008179) [54] and *Gr1*^*a1Neu*^ (MGI: 1857772) [55,56] alleles. All mice were kept on a mixed C57BL/6 genetic background. Mice were infected intranasally with 2.25 × 10^7^ PFU Ad5CMVCre (University of Iowa) to induce lung tumors under anesthesia as previously described [41]. Mice were collected 90 days post infection, lungs were inflated with formalin and fixed, and lungs were embedded in paraffin for analysis.

**Immunohistochemistry.** Paraffin-embedded tumor-bearing mouse lungs were sectioned, deparaffinized and rehydrated, followed by heat-mediated antigen retrieval with boiling 10 mmol/L citrate buffer (pH 6.0). Antibodies used for IHC are as follows: GSR (Santa Cruz Biotechnology, Cat # sc-133245, 1:100), TXNRD1 (Cell Signaling Technologies, Cat #15140S, 1:100), NQO1 (Sigma-Aldrich, Cat #HPA007308, 1:250), F4/80 (Cell Signaling Technologies, Cat# 70076, 1:250), Arg1 (Cell Signaling Technologies, Cat#93668, 1:1000), iNOS (Cell Signaling Technologies, Cat #681865, 1:100) and GSH (Virogen, Cat# 101A100, 1:100). Sections were incubated at 4 °C in primary antibody overnight. The ImmPRESS HRP (horseradish peroxidase) goat anti-rabbit kit (Vector Laboratories, RRID:AB_2631198) was used as directed by the manufacturer's instructions. For the mouse antibodies GSR and GSH, the M.O.M (Mouse-on-Mouse) ImmPRESS IgG HRP polymer kit (Vector Laboratories, RRID:AB_2336832) was used to reduce endogenous mouse Ig staining. DAB peroxidase (HRP) substrate (Vector Laboratories, SK-4105) was used, followed by counterstaining with hematoxylin (Vector Laboratories, H-3404). Slide scanning at 20 × was performed with an Aperio imager and the H-score of at least five representative regions/mouse was analyzed with QuPath software [69]. The Axio Lab A.1 microscope was used to take representative images at x 40 (Carl Zeiss Microimaging Inc.)

**Tumor grading analysis and histology.** Lung tumors were manually graded using a previously published protocol [54]. Tumor number was determined by normalizing the total tumor number per mouse H&E section to the lung area. The distribution of tumor grades among genotypes was determined by dividing the number of tumors per grade by the number of tumors per mouse. Tumor burden per grade was obtained by dividing the area of the lung occupied by tumors of a specific grade by the total lung area.

**Quantification and statistical analysis.** GraphPad Prism9 software was used to perform statistical tests as indicated (∗p < 0.05; ∗∗p < 0.01; ∗∗∗p < 0.001; ∗∗∗∗*p* < 0.0001; ns, non-significant). All data are expressed as mean ± SD. A ROUT outlier test was performed and one data point corresponding to a Nrf2^+/+^ TXNRD1 GSR KO tumor in Supplementary Fig. 3E–F for grade 1 burden was excluded.

## CRediT authorship contribution statement

**Amanda M. Sherwood:** Conceptualization, Investigation, Methodology, Writing – original draft. **Basma A. Yasseen:** Investigation, Methodology, Writing – review & editing. **Janine M. DeBlasi:** Investigation, Writing – review & editing. **Samantha Caldwell:** Investigation, Writing – review & editing. **Gina M. DeNicola:** Conceptualization, Funding acquisition, Methodology, Supervision, Writing – original draft.

## Declaration of competing interest

The authors declare that they have no known competing financial interests or personal relationships that could have appeared to influence the work reported in this paper.

## References

[1] DeBlasi J.M., DeNicola G.M. (2020). Dissecting the Crosstalk between NRF2 signaling and metabolic processes in cancer. Cancers (Basel).

[2] Kensler T.W., Wakabayash N., Biswal S. (2007). Cell survival responses to environmental stresses via the Keap1-Nrf2-ARE pathway. Annu. Rev. Pharmacol..

[3] He F., Ru X., Wen T. (2020). NRF2, a transcription factor for stress response and beyond. Int. J. Mol. Sci..

[4] Itoh K. (1999). Keap1 represses nuclear activation of antioxidant responsive elements by Nrf2 through binding to the amino-terminal Neh2 domain. Genes Dev..

[5] Dayalan Naidu S., Dinkova-Kostova A.T. (2020). KEAP1, a cysteine-based sensor and a drug target for the prevention and treatment of chronic disease. Open Biol.

[6] Hirotsu Y. (2012). Nrf2-MafG heterodimers contribute globally to antioxidant and metabolic networks. Nucleic Acids Res..

[7] Hayes J.D., Dinkova-Kostova A.T. (2014). The Nrf2 regulatory network provides an interface between redox and intermediary metabolism. Trends Biochem. Sci..

[8] Wu K.C., Cui J.Y., Klaassen C.D. (2012). Effect of graded Nrf2 activation on phase-I and -II drug metabolizing enzymes and transporters in mouse liver. PLoS One.

[9] He F., Antonucci L., Karin M. (2020). NRF2 as a regulator of cell metabolism and inflammation in cancer. Carcinogenesis.

[10] Mitsuishi Y. (2012). Nrf2 redirects glucose and glutamine into anabolic pathways in metabolic reprogramming. Cancer Cell.

[11] Kang Y.P. (2019). Cysteine dioxygenase 1 is a metabolic liability for non-small cell lung cancer. Elife.

[12] Wu K.C., Cui J.Y., Klaassen C.D. (2011). Beneficial role of Nrf2 in regulating NADPH generation and consumption. Toxicol. Sci..

[13] Ludtmann M.H.R., Angelova P.R., Zhang Y., Abramov A.Y., Dinkova-Kostova A.T. (2014). Nrf2 affects the efficiency of mitochondrial fatty acid oxidation. Biochem. J..

[14] Romero R. (2017). Keap1 loss promotes Kras-driven lung cancer and results in dependence on glutaminolysis. Nat Med.

[15] Reisman S.A., Yeager R.L., Yamamoto M., Klaassen C.D. (2009). Increased Nrf2 activation in livers from keap1-knockdown mice increases expression of cytoprotective genes that detoxify electrophiles more than those that detoxify reactive oxygen species. Toxicol. Sci..

[16] Harvey C.J. (2009). Nrf2-regulated glutathione recycling independent of biosynthesis is critical for cell survival during oxidative stress. Free Radical Bio Med.

[17] Erickson A.M., Nevarea Z., Gipp J.J., Mulcahy R.T. (2002). Identification of a variant antioxidant response element in the promoter of the human glutamate-cysteine ligase modifier subunit gene - revision of the ARE consensus sequence. J. Biol. Chem..

[18] Deponte M. (2013). Glutathione catalysis and the reaction mechanisms of glutathione-dependent enzymes. Bba-Gen Subjects.

[19] Moinova H.R., Mulcahy R.T. (1999). Up-regulation of the human γ-glutamylcysteine synthetase regulatory subunit gene involves binding of Nrf-2 to an electrophile responsive element. Biochem Bioph Res Co.

[20] Hawkes H.J.K., Karlenius T.C., Tonissen K.F. (2014). Regulation of the human thioredoxin gene promoter and its key substrates: a study of functional and putative regulatory elements. Bba-Gen Subjects.

[21] Sakurai A. (2005). Transcriptional regulation of thioredoxin reductase 1 expression by cadmium in vascular endothelial cells: role of NF-E2-related factor-2. J. Cell. Physiol..

[22] Osburn W.O. (2006). Nrf2 regulates an adaptive response protecting against oxidative damage following diquat-mediated formation of superoxide anion. Arch. Biochem. Biophys..

[23] Salazar M., Rojo A.I., Velasco D., de Sagarra R.M., Cuadrado A. (2006). Glycogen synthase kinase-3β inhibits the xenobiotic and antioxidant cell response by direct phosphorylation and nuclear exclusion of the transcription factor Nrf2. J. Biol. Chem..

[24] Toppo S., Flohé L., Ursini F., Vanin S., Maiorino M. (2009). Catalytic mechanisms and specificities of glutathione peroxidases: variations of a basic scheme. Bba-Gen Subjects.

[25] Flohé L., Toppo S., Cozza G., Ursini F. (2011). A comparison of thiol peroxidase mechanisms. Antioxid Redox Sign.

[26] Rhee S.G., Woo H.A., Kil I.S., Bae S.H. (2012). Peroxiredoxin functions as a peroxidase and a regulator and sensor of local peroxides. J. Biol. Chem..

[27] Lu J., Holmgren A. (2014). The thioredoxin antioxidant system. Free Radical Bio Med.

[28] Sun Q.A. (1999). Redox regulation of cell signaling by selenocysteine in mammalian thioredoxin reductases. J. Biol. Chem..

[29] Holmgren A. (2000). Antioxidant function of thioredoxin and glutaredoxin systems. Antioxid Redox Sign.

[30] Holmgren A., Bjornstedt M. (1995). Thioredoxin and thioredoxin reductase. Method Enzymol.

[31] Bannai S. (1986). Exchange of cystine and glutamate across plasma-membrane of human-fibroblasts. J. Biol. Chem..

[32] Mandal P.K. (2010). System x_c_- and thioredoxin reductase 1 cooperatively rescue glutathione deficiency. J. Biol. Chem..

[33] Espinosa B., Arnér E.S.J. (2019). Thioredoxin-related protein of 14 kDa as a modulator of redox signalling pathways. Brit J Pharmacol.

[34] Pader I. (2014). Thioredoxin-related protein of 14 kDa is an efficient L-cystine reductase and S-denitrosylase. P Natl Acad Sci USA.

[35] Jiang X.J., Stockwell B.R., Conrad M. (2021). Ferroptosis: mechanisms, biology and role in disease. Nat Rev Mol Cell Bio.

[36] Ingold I. (2018). Selenium utilization by GPX4 is required to prevent hydroperoxide-induced ferroptosis. Cell.

[37] Veal E.A., Day A.M., Morgan B.A. (2007). Hydrogen peroxide sensing and signaling. Mol Cell.

[38] Kamata H., Hirata H. (1999). Redox regulation of cellular signalling. Cell. Signal..

[39] Zhang Y. (2020). Redox regulation of tumor suppressor PTEN in cell signaling. Redox Biol..

[40] Ghezzi P. (2005). Regulation of protein function by glutathionylation. Free Radical Res.

[41] DeBlasi J.M. (2023). Distinct Nrf2 signaling thresholds mediate lung tumor initiation and progression. Cancer Res..

[42] Witherspoon J.G. (2024). Mutant Nrf2(E79Q) enhances the promotion and progression of a subset of oncogenic Ras keratinocytes and skin tumors. Redox Biol..

[43] Takahashi J. (2024). Differential squamous cell fates elicited by NRF2 gain of function versus KEAP1 loss of function. Cell Rep..

[44] Li C. (2021). Quantitative in vivo analyses reveal a complex pharmacogenomic landscape in lung adenocarcinoma. Cancer Res..

[45] Foggetti G. (2021). Genetic determinants of EGFR-driven lung cancer growth and therapeutic response in vivo. Cancer Discov..

[46] Best S.A. (2018). Synergy between the KEAP1/NRF2 and PI3K pathways drives non-small-cell lung cancer with an altered immune microenvironment. Cell Metab..

[47] Best S.A. (2019). Distinct initiating events underpin the immune and metabolic heterogeneity of Kras-mutant lung adenocarcinoma. Nat. Commun..

[48] Hayashi M. (2020). Microenvironmental activation of Nrf2 restricts the progression of nrf2-activated malignant tumors. Cancer Res..

[49] Jeong Y. (2017). Role of KEAP1/NRF2 and TP53 mutations in lung squamous cell carcinoma development and radiation resistance. Cancer Res..

[50] Singh A. (2021). NRF2 activation promotes Aggressive lung cancer and associates with poor clinical outcomes. Clin. Cancer Res..

[51] Lignitto L. (2019). Nrf2 activation promotes lung cancer metastasis by inhibiting the degradation of Bach1. Cell.

[52] Foggetti G. (2021). Genetic determinants of EGFR-driven lung cancer growth and therapeutic response. Cancer Discov..

[53] Rogers Z.N. (2018). Mapping the in vivo fitness landscape of lung adenocarcinoma tumor suppression in mice. Nat. Genet..

[54] Jackson E.L. (2001). Analysis of Lung Tumor Initiation and Progression Using Conditional Expression of Oncogenic K-ras. Gene Dev..

[55] Rogers L.K. (2004). Analyses of glutathione reductase hypomorphic mice indicate a genetic knockout. Toxicol. Sci..

[56] Pretsch W. (1999). Glutathione reductase activity deficiency in homozygous mice does not cause haemolytic anaemia. Genet. Res..

[57] Bondareva A.A. (2007). Effects of thioredoxin reductase-1 deletion on embryogenesis and transcriptome. Free Radical Bio Med.

[58] Harris I.S. (2015). Glutathione and thioredoxin antioxidant pathways synergize to drive cancer initiation and progression (vol 27, pg 211, 2015). Cancer Cell.

[59] Smith J.A. (2018). Regulation of cytokine production by the unfolded protein response; implications for infection and autoimmunity. Front. Immunol..

[60] Poet G.J. (2017). Cytosolic thioredoxin reductase 1 is required for correct disulfide formation in the ER. Embo J.

[61] Conroy L.R., Hawkinson T.R., Young L.E.A., Gentry M.S., Sun R.C. (2021). Emerging roles of N-linked glycosylation in brain physiology and disorders. Trends Endocrinol Metab.

[62] Nishinaka Y., Nakamura H., Yodoi J. (2002). Thioredoxin cytokine action. Protein Sensors and Reactive Oxygen Species, Pt a, Selenoproteins and Thioredoxin.

[63] Rubartelli A., Bajetto A., Allavena G., Wollman E., Sitia R. (1992). Secretion of thioredoxin by normal and neoplastic-cells through a leaderless secretory pathway. J. Biol. Chem..

[64] Bertini R. (1999). Thioredoxin, a redox enzyme released in infection and inflammation, is a unique chemoattractant for neutrophils, monocytes, and T cells. J. Exp. Med..

[65] Eriksson S., Prigge J.R., Talago E.A., Arner E.S., Schmidt E.E. (2015). Dietary methionine can sustain cytosolic redox homeostasis in the mouse liver. Nat. Commun..

[66] Yoon S.J. (2023). Comprehensive metabolic tracing reveals the origin and catabolism of cysteine in mammalian tissues and tumors. Cancer Res..

[67] Weiss Sadan T. (2023). NRF2 activation induces NADH-reductive stress, providing a metabolic vulnerability in lung cancer. Cell Metab..

[68] Sayin V.I. (2017). Activation of the NRF2 antioxidant program generates an imbalance in central carbon metabolism in cancer. Elife.

[69] Bankhead P., Loughrey M.B., Fernández J.A., QuPath (2017). Open source software for digital pathology image analysis. Sci. Rep..

