# Supplementary material for: Distinct roles for the thioredoxin and glutathione antioxidant systems in Nrf2-Mediated lung tumor initiation and progression
Source: Redox Biol. 2025 Apr 30;83:103653. doi: 10.1016/j.redox.2025.103653 (PMC12133717; doi:10.1016/j.redox.2025.103653)
Supplement: Multimedia component 1 [file mmc1.pdf]

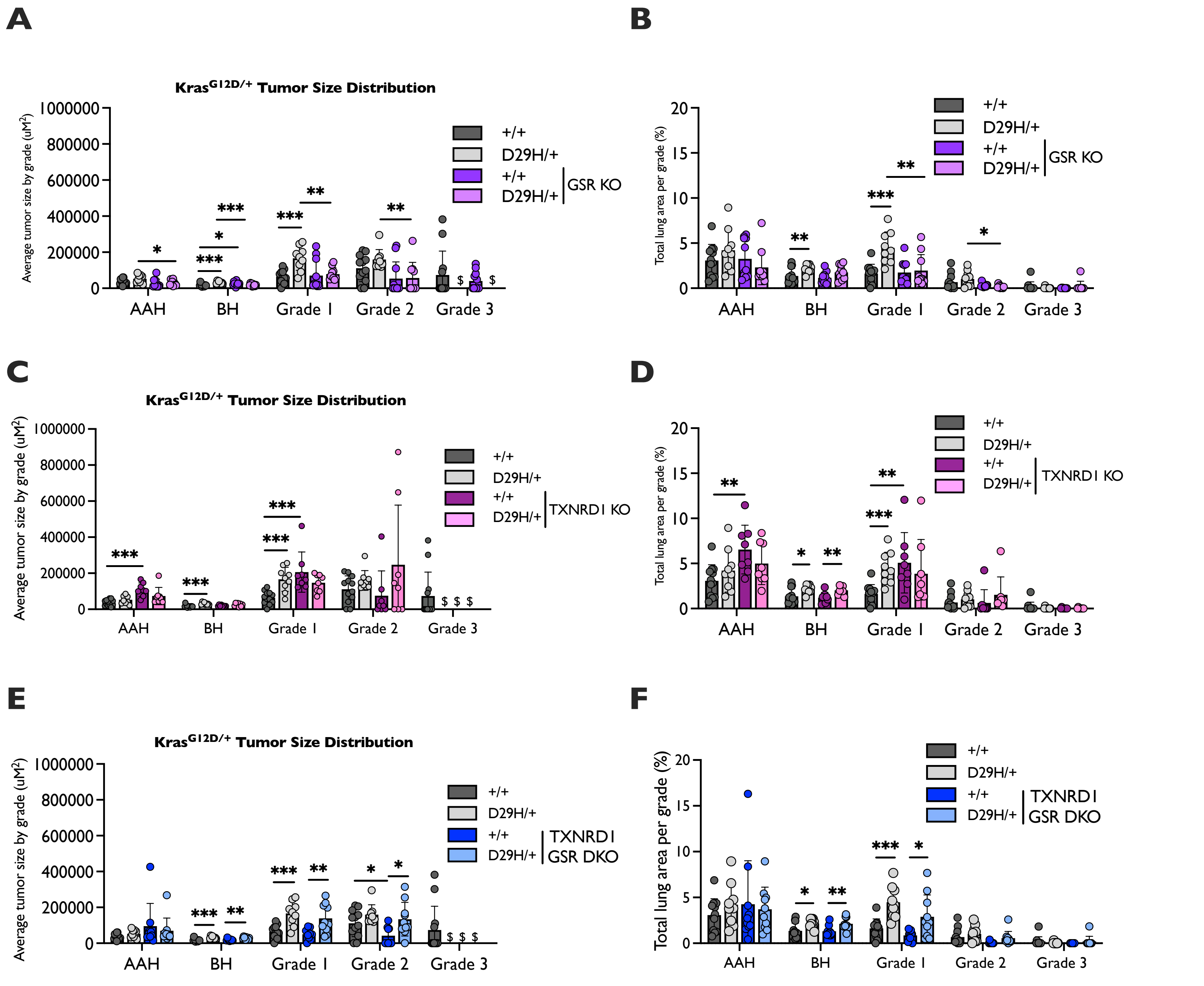

Supplementary Figure 1. Lung tumor size analysis. Distribution of lung tumor size by grade across Kras$^{G12D/+}$ and Kras$^{G12D/+;}$ Nrf2$^{D29H/+}$ mice for **(A)** GSR KO mice (n = 11 for Nrf2$^{+/+}$; *n* = 10 for Nrf2$^{D29H/+}$) **(C)** TXNRD1 KO mice (n = 8 for Nrf2$^{+/+}$; *n* = 8 for Nrf2$^{D29H/+}$), and **(E)** GSR/TXNRD1 KO mice (n = 10 for Nrf2$^{+/+}$ and Nrf2$^{D29H/+}$), compared to GSR/TXNRD1 WT mice (n = 12 for Nrf2$^{+/+}$; *n* = 9 for Nrf2$^{D29H/+}$, included in graphs in **A**, **C** and **E**). Fraction of lung tumor burden by grade (lung tumor area per grade/total lung area) for **(B)** GSR KO mice (n = 11 for Nrf2$^{+/+}$; *n* = 10 for Nrf2$^{D29H/+}$), **(D)** TXNRD1 KO mice (n = 8 for Nrf2$^{+/+}$; *n* = 8 for Nrf2$^{D29H/+}$), and **(F)** GSR/TXNRD1 KO mice (n = 10 for Nrf2$^{+/+}$ and Nrf2$^{D29H/+}$), compared to GSR/TXNRD1 WT mice (n = 12 for Nrf2$^{+/+}$; *n* = 9 for Nrf2$^{D29H/+}$, included in graphs in **B**, **D** and **F**). *$p<0.05$, **$p<0.01$, ***$p<0.001$, ****$p<0.0001$ (unpaired t-test with Holm–Sidak multiple comparisons test). $ = fewer than three tumors detected across all mice. AAH, alveolar adenomatous hyperplasia; BH, bronchiolar hyperplasia.
