# Supplementary material for: Distinct roles for the thioredoxin and glutathione antioxidant systems in Nrf2-Mediated lung tumor initiation and progression
Source: Redox Biol. 2025 Apr 30;83:103653. doi: 10.1016/j.redox.2025.103653 (PMC12133717; doi:10.1016/j.redox.2025.103653)
Supplement: Multimedia component 2 [file mmc2.pdf]

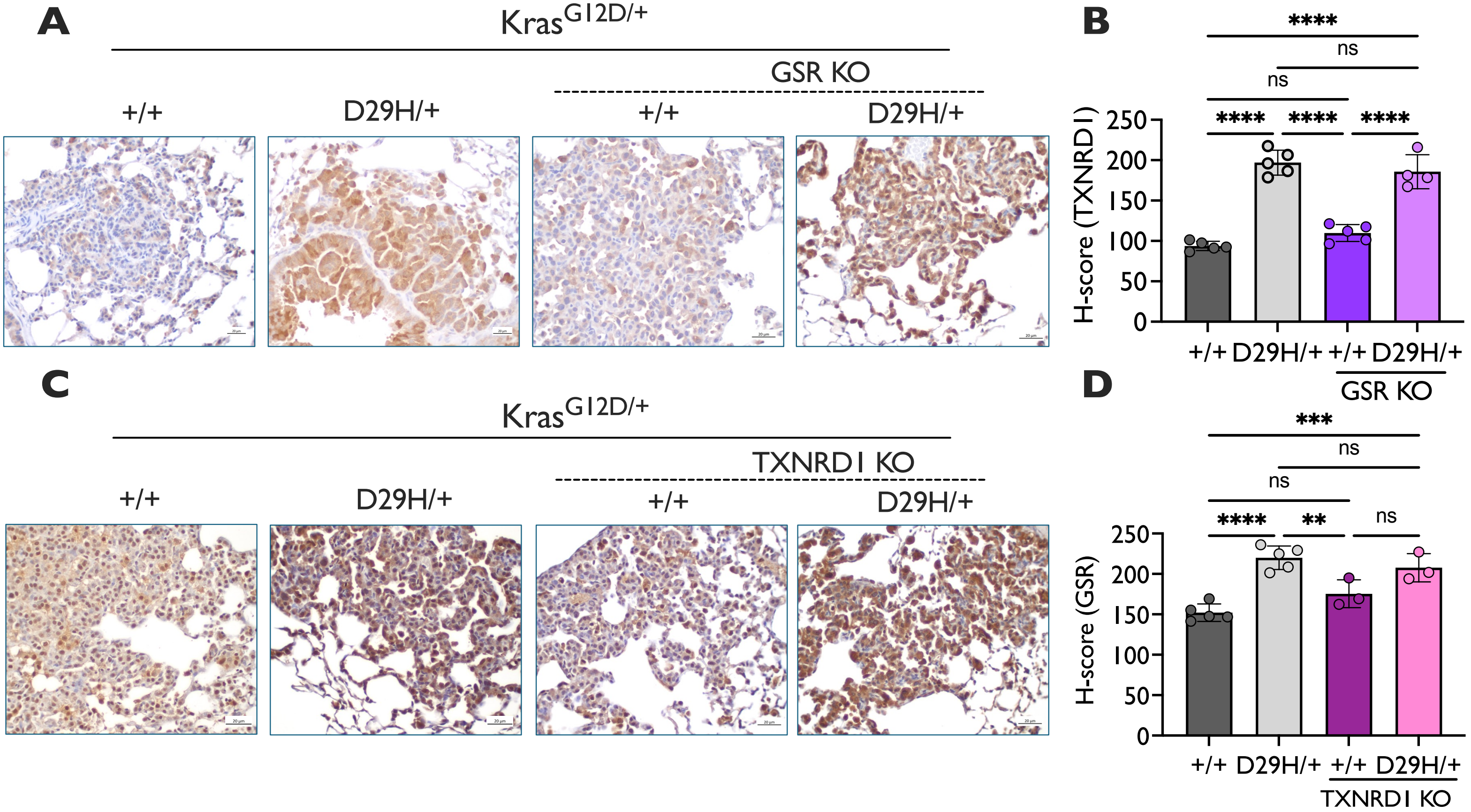

Supplementary Figure 2. GSR or TXNRD1 KO does not increase GSR or TXNRD1 expression. (A) Representative TXNRD1 IHC staining of Nrf2 +/+ and D29H/+ tumors that are GSR WT or GSR KO and **(B)** quantitative H-score analysis for TXNRD1 staining. ****p<0.0001, ns: non-significant (one-way ANOVA with Tukey's multiple comparison's test). n = 5 for Nrf2$^{+/+}$ and Nrf2$^{D29H/+}$ GSR WT; Nrf2$^{+/+}$ GSR KO (*n* = 5); Nrf2$^{D29H/+}$ GSR KO (*n* = 4). Scale bars, 20 µm. **(C)** Representative GSR immunohistochemical (IHC) staining of Nrf2 +/+ and D29H/+ tumors that are TXNRD1 WT or TXNRD1 KO and **(D)** quantitative H-score analysis for GSR staining. **p<0.01, ***p<0.001, ****p<0.0001, ns: non-significant (one-way ANOVA with Tukey's multiple comparison's test). n = 5 for Nrf2$^{+/+}$ and Nrf2$^{D29H/+}$ TXNRD1 WT; *n* = 3 for Nrf2$^{+/+}$ and Nrf2$^{D29H/+}$ TXNRD1 KO. Scale bars, 20 µm.
