# Supplementary material for: Distinct roles for the thioredoxin and glutathione antioxidant systems in Nrf2-Mediated lung tumor initiation and progression
Source: Redox Biol. 2025 Apr 30;83:103653. doi: 10.1016/j.redox.2025.103653 (PMC12133717; doi:10.1016/j.redox.2025.103653)
Supplement: Multimedia component 3 [file mmc3.pdf]

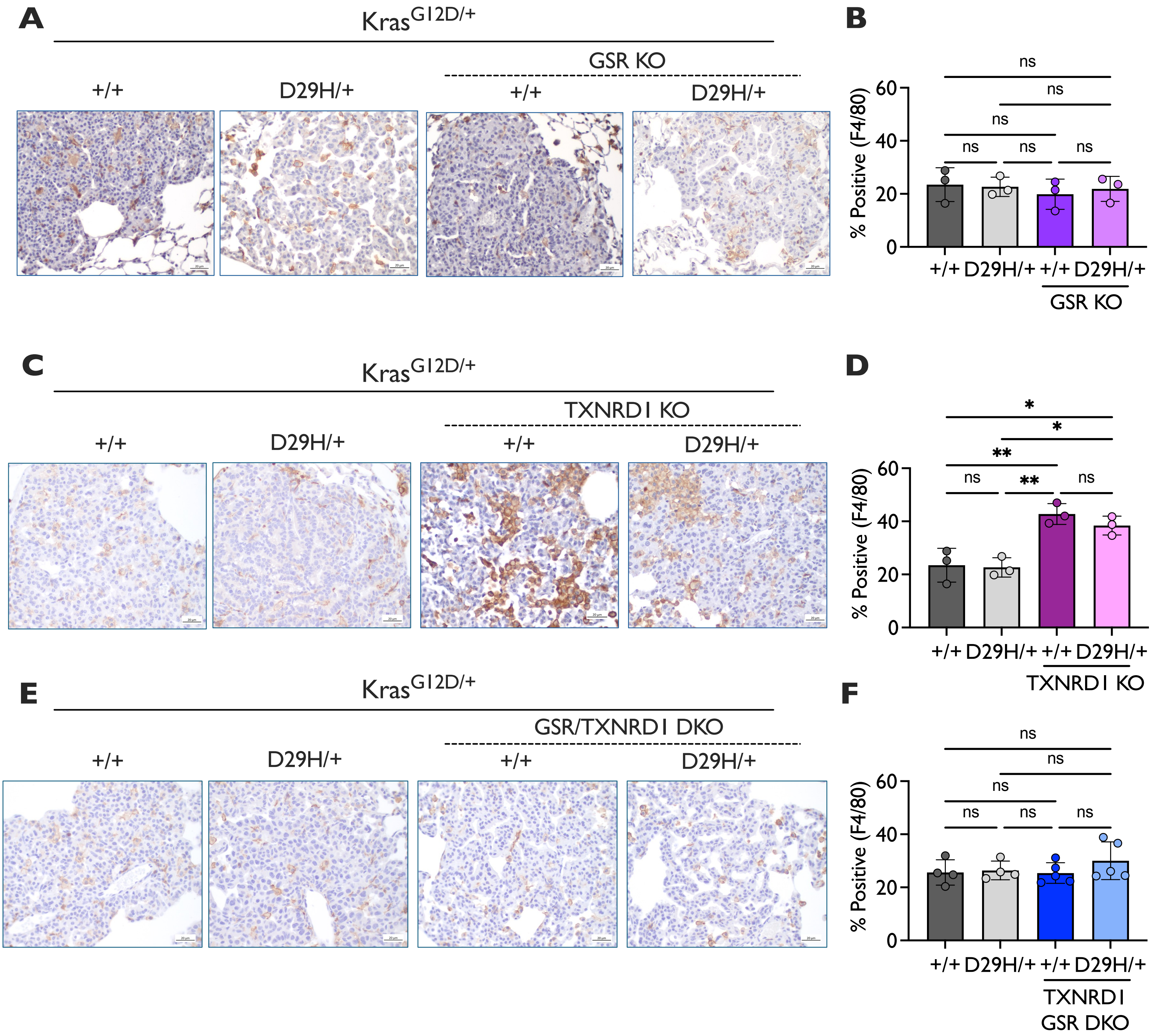

Supplementary Figure 3. Macrophage infiltration is increased in TXNRD1 KO tumors. Representative IHC staining for the macrophage marker F4/80 in $Nrf2^{+/+}$ and $Nrf2^{D29H/+}$ tumors that are **(A)** GSR WT or GSR KO **(C)** TXNRD1 WT or TXNRD1 KO and **(E)** GSR/TXNRD1 WT or GSR/TXNRD1 KO. Percentage of F4/80 positive cells per tumor for (**B)** GSR KO mice (n = 3 for $Nrf2^{+/+}$ and $Nrf2^{D29H/+}$), **(D)** TXNRD1 KO mice (n = 3 for $Nrf2^{+/+}$ and $Nrf2^{D29H/+}$), and **(F)** GSR/TXNRD1 KO mice (n = 5 for $Nrf2^{+/+}$ and $Nrf2^{D29H/+}$), compared to GSR/TXNRD1 WT mice (n = 3 for $Nrf2^{+/+}$ and $Nrf2^{D29H/+}$, included in graphs in **B** and **D; ** n = 4 for $Nrf2^{+/+}$ and $Nrf2^{D29H/+}$ in graph **F**). *$p<0.05$, **$p<0.01$, ns: non-significant (one-way ANOVA with Tukey's multiple comparison's test).
