# Supplementary material for: Distinct roles for the thioredoxin and glutathione antioxidant systems in Nrf2-Mediated lung tumor initiation and progression
Source: Redox Biol. 2025 Apr 30;83:103653. doi: 10.1016/j.redox.2025.103653 (PMC12133717; doi:10.1016/j.redox.2025.103653)
Supplement: Multimedia component 4 [file mmc4.pdf]

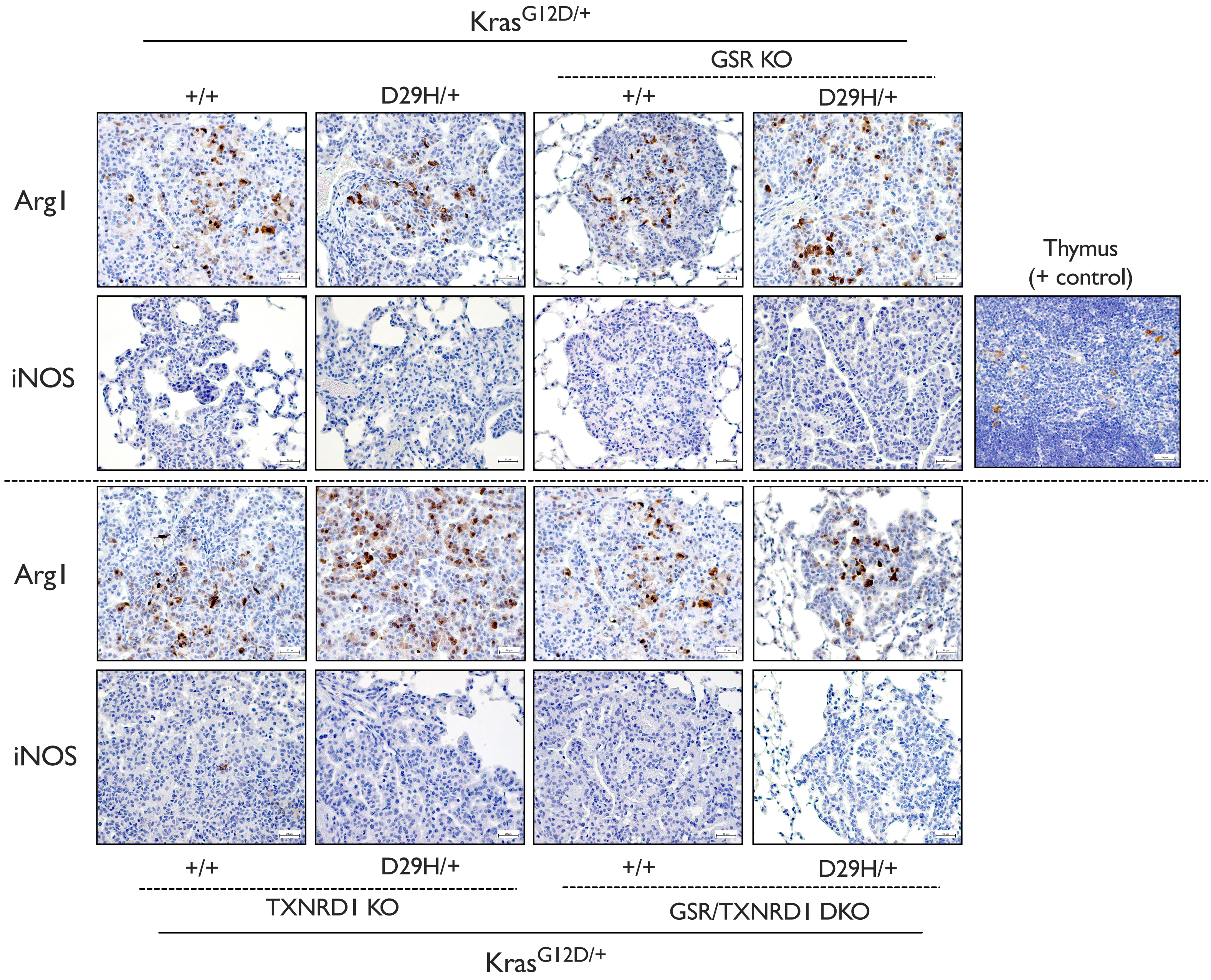

Supplementary Figure 4. Macrophages are Arg1 positive. Representative IHC staining for Arg1 and iNOS in $Nrf2^{+/+}$ and $Nrf2^{D29H/+}$ tumors that are WT, GSR KO, TXNRD1 KO or GSR/TXNRD1 KO. Images are representative of 5 individual mice per genotype. Thymus is included as a positive control for iNOS. Scale bars, 20 µm.
