# Supplementary material for: Distinct roles for the thioredoxin and glutathione antioxidant systems in Nrf2-Mediated lung tumor initiation and progression
Source: Redox Biol. 2025 Apr 30;83:103653. doi: 10.1016/j.redox.2025.103653 (PMC12133717; doi:10.1016/j.redox.2025.103653)
Supplement: Multimedia component 5 [file mmc5.pdf]

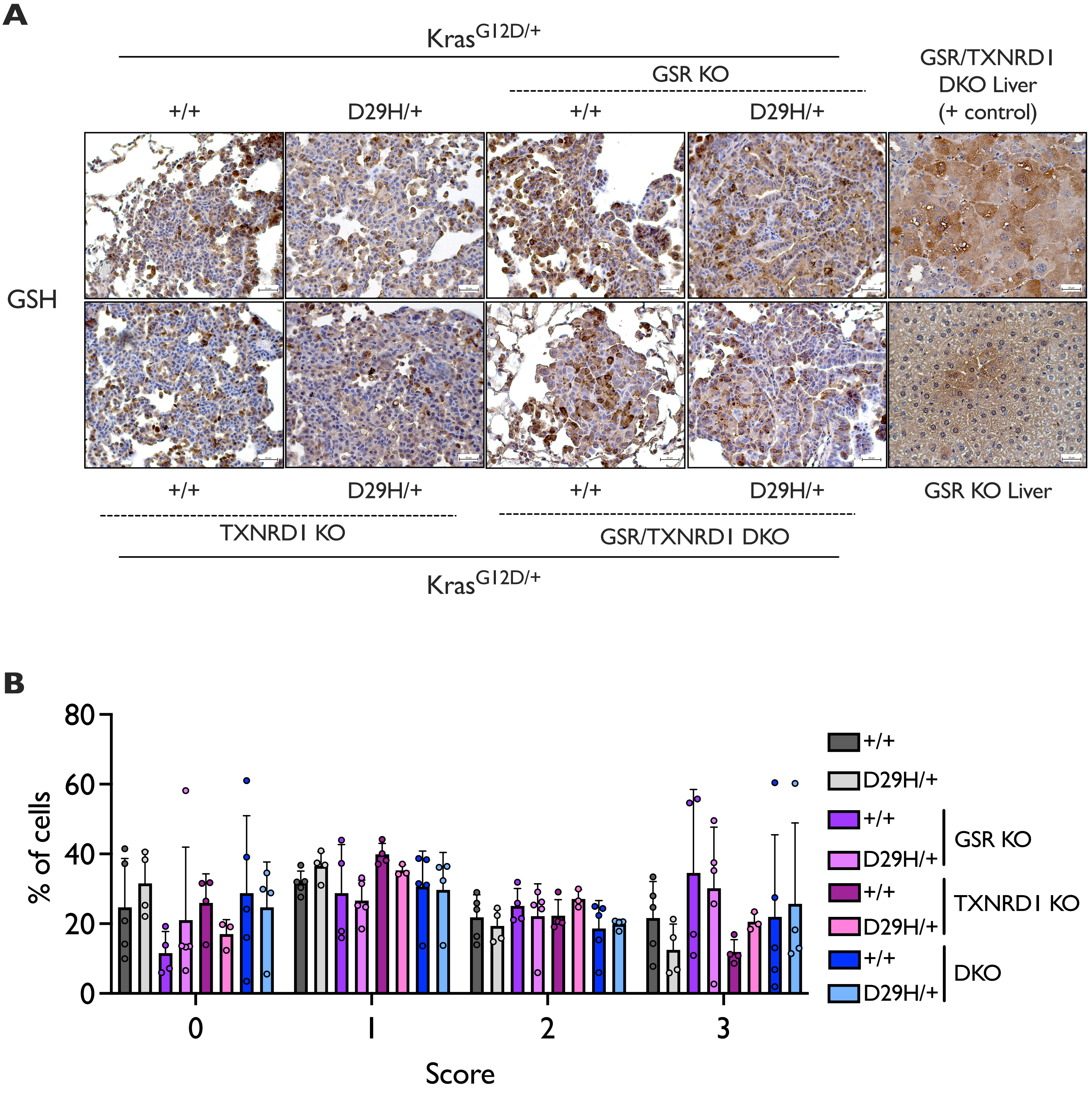

Supplementary Figure 5. Lack of protein glutathione adduct accumulation upon GSR and/ or TXNRD1 deletion. (A) Representative IHC staining for protein glutathione adducts (GSH) in Nrf2$^{+/+}$ and Nrf2$^{D29H/+}$ tumors that are WT, GSR KO, TXNRD1 KO or GSR/TXNRD1 KO. Images are representative of 5 individual mice per genotype. Scale bars, 20 μm. **(B)** Percentage of cells based on GSH staining intensity (0=no staining, 1=light, 2=medium, 3=dark). Scoring was performed by QuPath using the same threshold across all images. p=ns: non-significant (one-way ANOVA with Tukey's multiple comparison's test).
